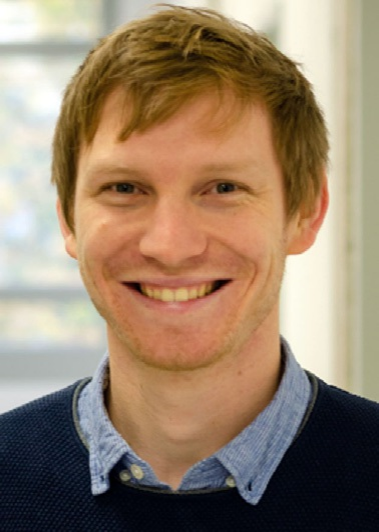# Recent Advances in Enantioselective Desymmetrizations of Prochiral Oxetanes

**DOI:** 10.1002/chem.202004923

**Published:** 2021-02-02

**Authors:** Alexander Sandvoß, Johannes M. Wiest

**Affiliations:** ^1^ Department Chemie Johannes Gutenberg Universität Mainz Duesbergweg 10–14 55128 Mainz Germany; ^2^ Organisch-Chemisches Institut Westfälische Wilhelms-Universität Münster Corrensstrasse 36 48149 Münster Germany

**Keywords:** asymmetric synthesis, desymmetrization, oxetane, oxygen heterocycles, strained molecules

## Abstract

Strain relief of oxetanes offers a plethora of opportunities for the synthesis of chiral alcohols and ethers. In this context, enantioselective desymmetrization has been identified as a powerful tool to construct molecular complexity and this has led to the development of elegant strategies on the basis of transition metal, Lewis acid, and Brønsted acid catalysis. This review highlights recent examples that harness the inherent reactivity of prochiral oxetanes and offers an outlook on the immense possibilities for synthetic application.

## Graphical abstract

**Prochiral oxetanes** are excellent candidates for the synthesis of chiral alcohols and ethers via enantioselective desymmetrization. Recent methods relying on transition metal, Lewis acid, and Brønsted acid catalysis allow access to a remarkably diverse set of compounds, which are highlighted and put into context.

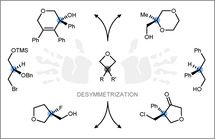

## 1 Introduction

**Scheme 1:**
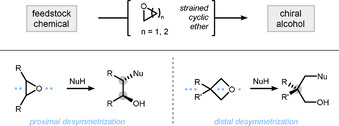
Desymmetrization of cyclic ethers for the synthesis of chiral alcohols.

The strain embedded in small cyclic ethers has led to their wide application in the synthesis of chiral alcohols.^[1]^ An elegant way to introduce asymmetry is through the synthesis of a prochiral cyclic ether followed by a subsequent desymmetrization step. In contrast to oxiranes (epoxides),^[2]^ desymmetrization of oxetanes can occur at a distant, sterically accessible position within the molecule, which offers a unique opportunity for the synthesis of chiral alcohols, especially those bearing β‐quaternary stereocenters (Scheme 1).

**Scheme 2:**
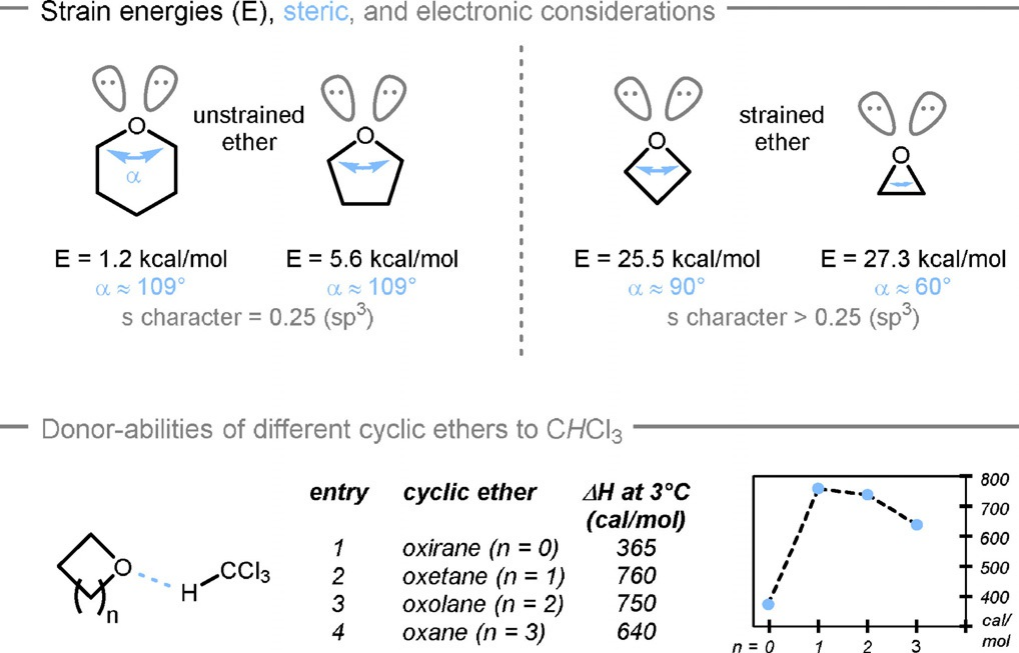
Physical properties of cyclic ethers.

Since the late 1980s numerous strategies for desymmetrization have been developed utilizing transition metal catalysis, organocatalysis, and enzymatic methods.^[3]^ Caused by their growing importance as bioisosters in medicinal chemistry, a plethora of methods for the synthesis of oxetanes came to fruition fueling the development of new desymmetrization reactions in recent years.^[4]^ Due to their high ring‐strain,^[5]^ oxetanes are prone to similar opening and atom‐insertion reactions as oxiranes (Scheme 2, top).^[6]^ Interestingly, the oxygen atom in the oxetane ring is surprisingly Lewis basic, which can be explained by two contradictory effects. Whereas a narrow C‐O‐C angle leaves the oxygen lone pair less sterically hindered, it also changes the hybridization of the ether's non‐bonding orbitals to higher s‐character.^[7]^ It has been shown experimentally through heat of mixing energies that oxetane comprises a favorable balance of the two effects (Scheme 2, bottom).^[8]^ This manifests in a superior electron donor ability compared to oxirane as well as oxolane (tetrahydrofuran) and oxane (tetrahydropyran). Similar trends have been observed for the binding to phenol,^[9]^ iodine,^[10]^ boron trifluoride,^[11]^ and the protonation with nitric acid.^[12]^ Accordingly, oxetanes are privileged structures for activation with Lewis acids or Brønsted acids, and are prone to undergo subsequent ring‐opening reactions with an appropriate nucleophile.[^1f^, ^13^] In addition, insertion in the C−O bonds is possible, albeit so far not in an asymmetric fashion.^[14]^

**Scheme 3:**
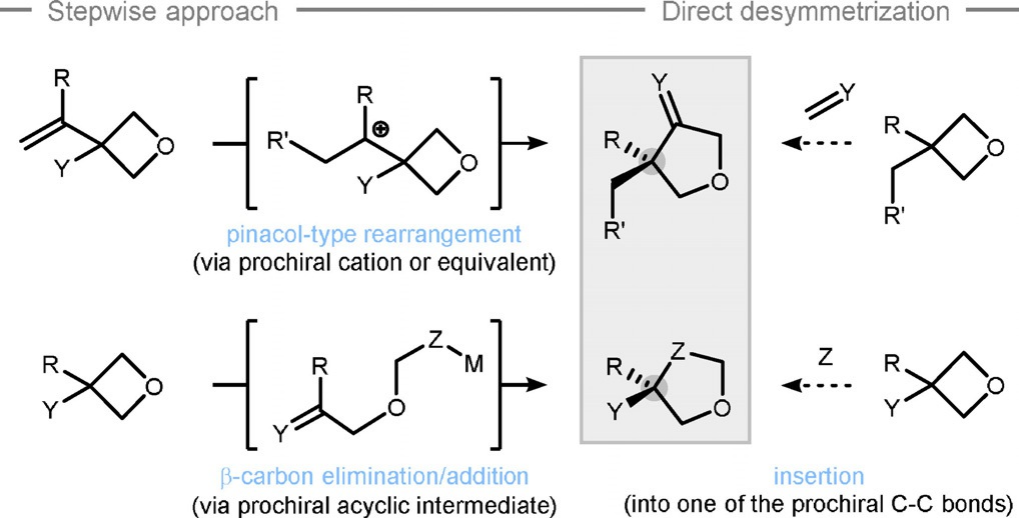
Strategies for remote oxetane desymmetrization.

Besides the common reactivity, the distorted C−C σ‐bonds of the oxetane ring^[15]^ offer opportunities to be activated by β‐carbon elimination, rearrangement, and bond‐insertion (Scheme 3). In these synthetic sequences, the two ether C−O σ bonds stay untouched and the reactivity of the oxetanes often resembles the one of the corresponding cyclobutanes.^[16]^ However, reaction development is affected by two major differences arising from the ether oxygen through inductive effects and competitive binding (due to the high Lewis basicity). Aside from that, no stereocenter can be created at the oxygen atom and current methods therefore focus on a stepwise desymmetrization approach via pinacol‐type rearrangement and β‐carbon elimination/addition (Scheme 3, left). The asymmetric direct insertion has so far not been achieved (Scheme 3, right). The products from these “remote” oxetane activations are highly diverse and synthetically useful, and clearly differentiate desymmetrizations of oxetanes from the related oxiranes.

In this review, current methods that achieve enantioselective desymmetrizations are presented based on the strategies discussed above. First, the focus will be laid on the breaking of the C−O bond by nucleophilic ring‐opening reactions arranged according to the type of nucleophile that is used. In the second section, the breaking of the remote C−C bond will be of interest and strategies involving transition metals will be discussed before a final conclusion and outlook will be drawn.

## 2 Nucleophilic Ring‐Opening Reactions

**Scheme 4:**
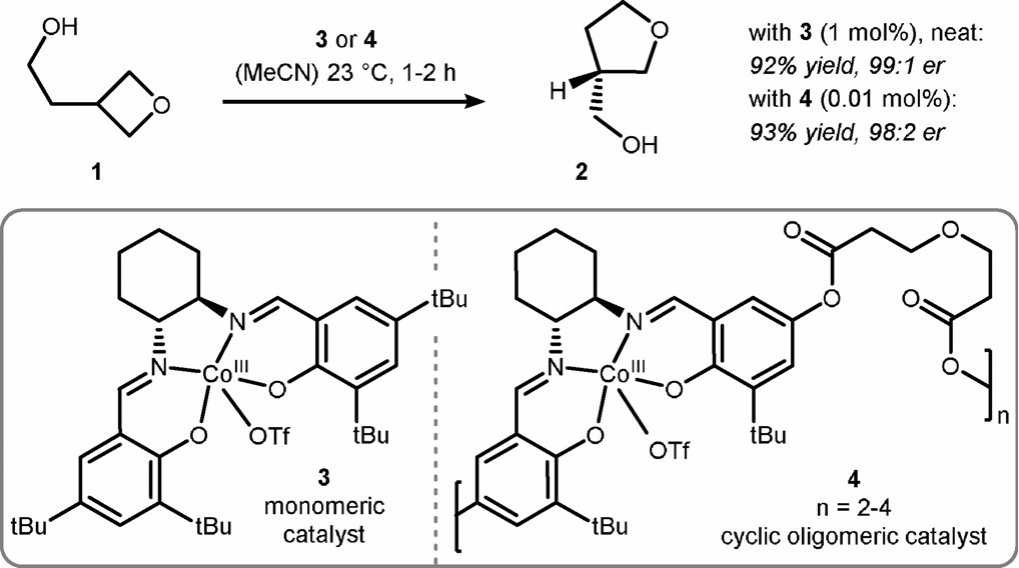
Jacobsen's Co^III^‐catalyzed intramolecular ring‐opening reaction towards oxolanes. Tf = trifluoromethanesulfonyl.

Oxetane ring‐opening reactions by oxygen nucleophiles are important for heterocyclic syntheses and were employed in numerous studies.^[17]^ Recently, Carreira et al. described an oxetane desymmetrization via the activation of the oxetane ring by addition of indium and boron‐based Lewis acids.^[18]^ Other types of Lewis acids have also been explored, such as Co^III^ in an asymmetric transformation reported by the Jacobsen group in 2009. Here, a ring‐opening reaction of oxetanol **1** to oxolane **2** catalyzed by Co^III^⋅salen **3** was described (Scheme 4).^[19]^ A cooperative bimetallic effect was found based on the superior reactivity of oligomeric Co^III^‐catalyst **4** compared to the monomeric counterpart **3**. Interestingly, this method also allowed the formation of quaternary stereocenters when 3,3‐disubstituted oxetanes were used. The driving force for this *trans*‐etherification can be found in the difference in strain energies between the oxolane and oxetane rings (∼20 kcal mol^−1^, cf. Scheme 2).

**Scheme 5:**
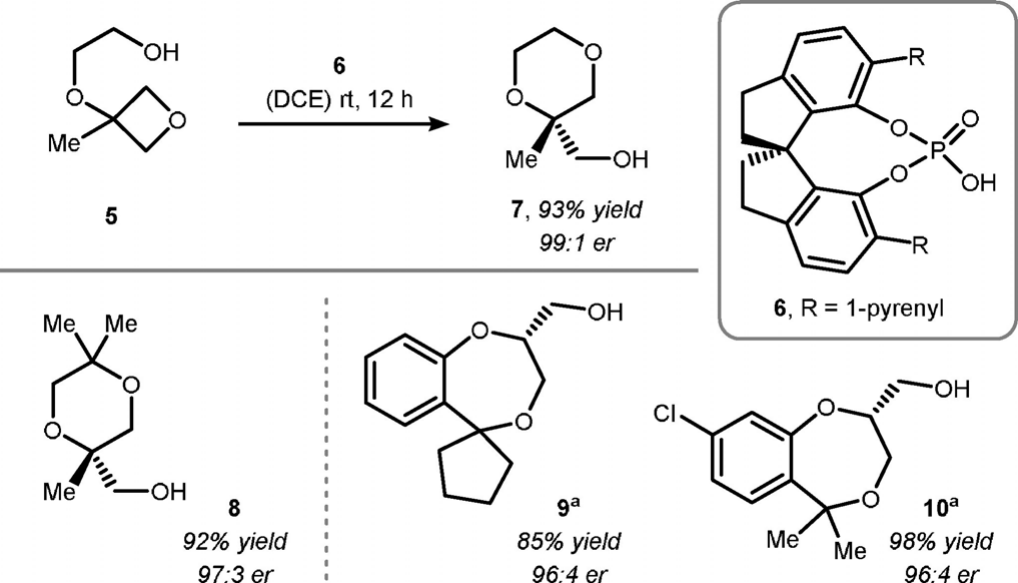
Sun's intramolecular Brønsted‐acid catalyzed desymmetrization of oxetanes. DCE = 1,2‐dichloroethane. a) Reaction run in PhMe with *ent*‐**6**.

**Figure 1:**
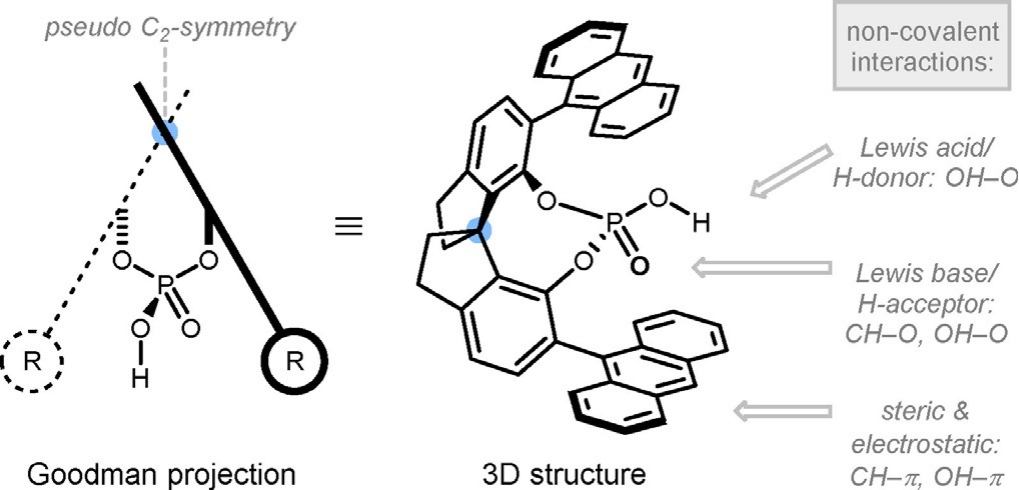
Goodman and 3D representation of common CPAs with points for substrate‐catalyst interactions.

Based on initial findings on Brønsted‐acid catalyzed ring‐opening reactions to lactones,^[20]^ the Sun group elaborated on oxygen‐based ring‐opening reactions utilizing chiral phosphoric acids (CPAs) for enantioinduction (Scheme 5, top).^[21]^ In their first communication, they reported oxetane ring‐opening of **5** by 1,1′‐spirobiindane‐7,7′‐diol (SPINOL) derived CPA **6** to give dioxane **7** in 99:1 enantiomeric ratio (er). Interestingly, the authors were also able to access sterically encumbered dioxanes such as **8** (Scheme 5, bottom). Even when the size of the nucleophile tether was extended, the yields and observed enantioselectivities stayed excellent. Later, the same group expanded the scope towards the enantioselective synthesis of 1,4‐benzodioxepines such as **9** and **10**.^[22]^ Mechanistically, CPA **6** was suspected to increase the electrophilicity of oxetane **5** through hydrogen‐bonding. The pseudo‐*C*_2_‐symmetric nature of catalyst **6** is enabling the high enantioselectivity though effective steric shielding by the large aryl units (see Figure 1 for a Goodman and 3D representation).

The initial postulation that the acid motif of the CPA has a dual role by activating both the oxetane and the nucleophile had to be revised due to recent theoretical calculations on the transition state geometry of this intramolecular process. It appears more likely that hydrogen‐bonding is activating the oxetane ring, and a noncovalent interaction between the π‐system of the CPA and the nucleophilic OH‐group is involved.^[23]^ This argument also explains the privileged role of large π‐systems as flanking groups (e.g. 9‐anthryl and 1‐pyrenyl) in these types of catalysts.

**Scheme 6:**
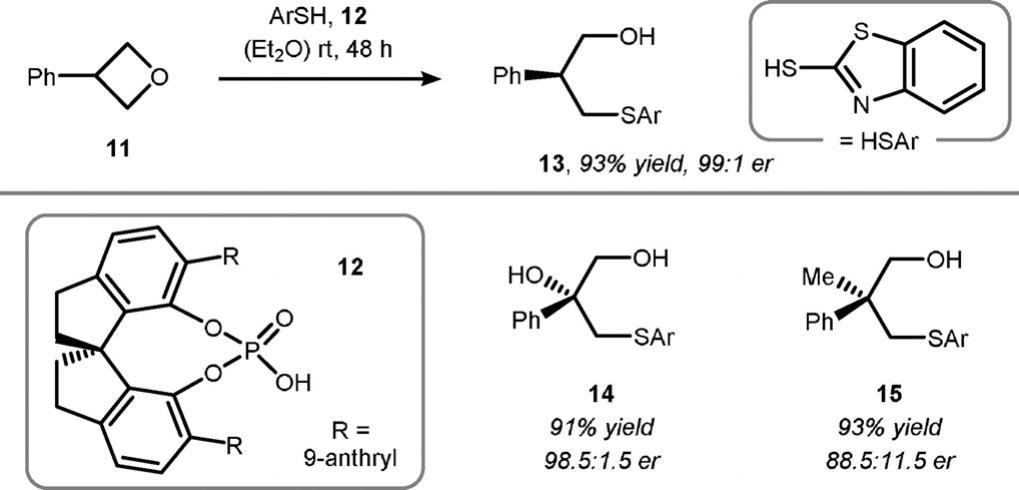
Sun's intermolecular ring‐opening reaction using sulfur nucleophiles.

In comparison to oxygen nucleophiles, sulfur compounds allow for intermolecular ring‐opening reactions to occur, presumably due to their superior nucleophilicity. In this regard, Sun et al. were able to desymmetrize 3‐phenyloxetane (**11**) with mercaptobenzothiazole and CPA **12** generating acyclic thioether **13** (Scheme 6).^[24]^ The possibility to access tertiary alcohols (e.g. **14**), quaternary all‐carbon stereocenters (e.g. **15**), and a number of useful functionalizations of the sulfur aryl group (e.g. Julia‐olefination^[25]^) highlight the synthetic utility of this protocol.

**Scheme 7:**
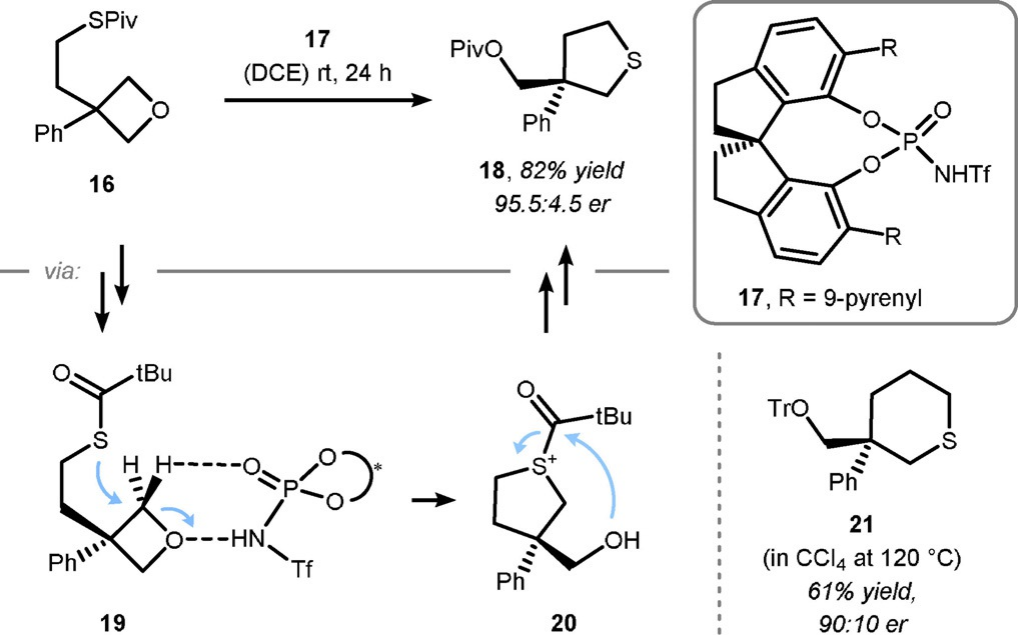
Houk and Sun's mechanistic rationale for the enantioselective desymmetrization of **16**. Piv = pivalate. Tr = triphenylmethyl.

As in prior examples, this transformation allows the control of stereocenters remote to the reaction site. The observed enantioselectivity was explained by the transition state geometry, which differs depending on the ability of the substituents to undergo hydrogen bonding to the catalyst, and the respective arrangement of its steric backbone (cf. Figure 1). Based on the aforementioned results, the Sun and Houk groups collaborated to study the mechanism of intramolecular ring‐opening reactions with protected oxetanol **16** as an exemplary substrate (Scheme 7).^[26]^ The influence of different CPA's was investigated in this study, but these acids were generally not effective in catalyzing the transformation, presumably due to their insufficient acidity. Hence, chiral phosphoramidate **17** having a higher acidity was identified. With this catalyst, the reaction proceeded in high yield and in an enantioselective fashion. The proposed mechanism entailing an intramolecular nucleophilic attack of the protected sulfur (intermediate **19**) and a subsequent intramolecular protecting group exchange (via **20**) is based on density functional theory (DFT) calculations and cross‐over experiments. Moreover, this reaction was tolerant to different substituents at the 3‐position of the oxetane ring and even allowed thiane **21** to be accessed when adjusting the protecting group.

**Scheme 8:**
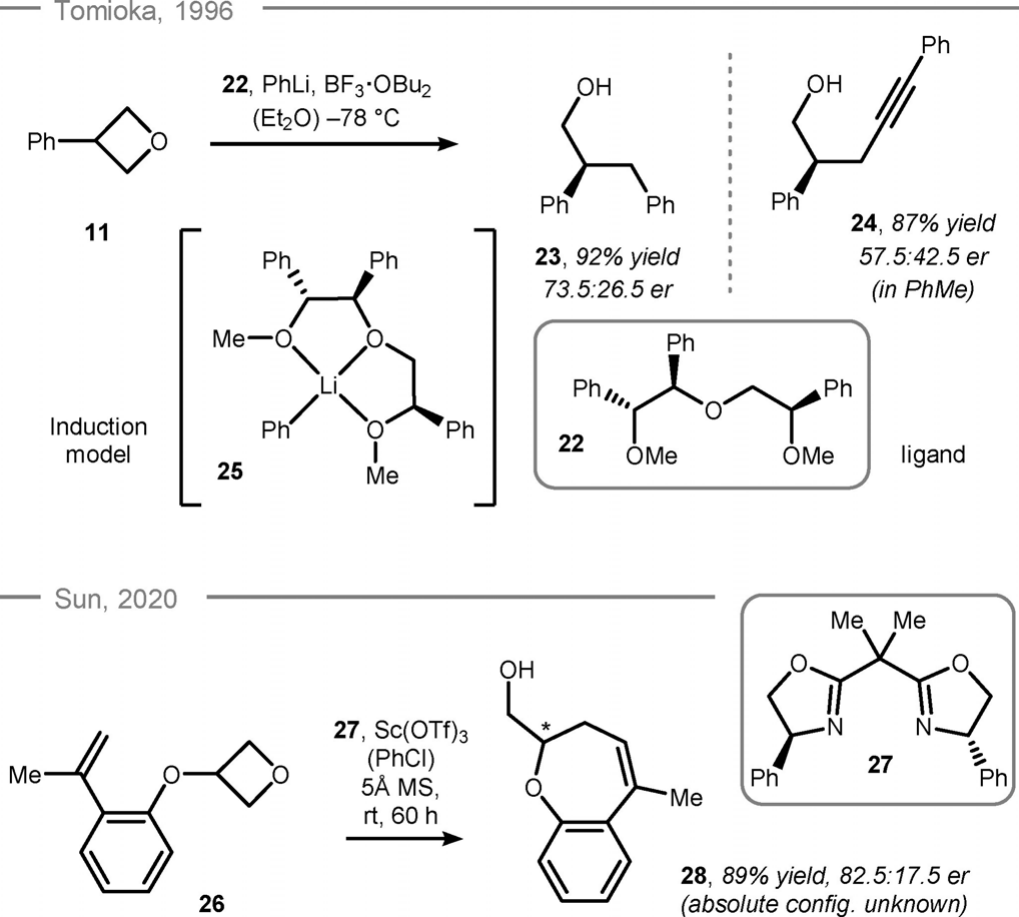
Top: Tomioka's carbon‐based ring‐opening. Bottom: Sun's intramolecular Prins‐type reaction. MS = molecular sieves.

Ring‐opening reactions with carbon nucleophiles such as alkyl lithiums or enolates are rare, even in a racemic fashion.^[27]^ In 1996, the first enantioselective desymmetrization using a lithiated carbon nucleophile was reported by Tomioka et al. In this study, 3‐phenyloxetane (**11**) was treated with stoichiometric amounts of boron trifluoride along with chiral ligand **22** (Scheme 8, top).^[28]^ It was postulated that polyether **22** coordinates the lithium ion in a rigid bicyclic structure (such as **25**), which directs the attack of the phenyl anion and thus acts as the origin of enantioselectivity for the formation of chiral alcohol **23** in 73.5:26.5 er. Interestingly, the enantiomeric excess was also influenced by the type of Lewis acid, with boron trifluoride being the best choice activating the oxygen atom of the cyclic ether before the nucleophilic attack. Different nucleophiles such as lithium phenylacetylide were viable and provided the corresponding product **24** in high yield, albeit moderate selectivity. This example shows the diversity of products that can be generated, but also highlights the limits of current protocols in terms of enantioselectivity. Promising results were observed in a very recent study by Sun et al. concerning soft carbon nucleophiles.^[29]^ While auxiliary directed silyl enol ether nucleophiles were successful in ring‐opening with moderate selectivity, a drastic improvement was witnessed on one example of an intramolecular Prins‐type reaction using chiral ligand **27** and a scandium Lewis acid (Scheme 8, bottom). Thus, conversion of oxetane **26** to dihydrooxepine **28** occurred in 89 % yield and a selectivity of 82.5:17.5 er.

**Scheme 9:**
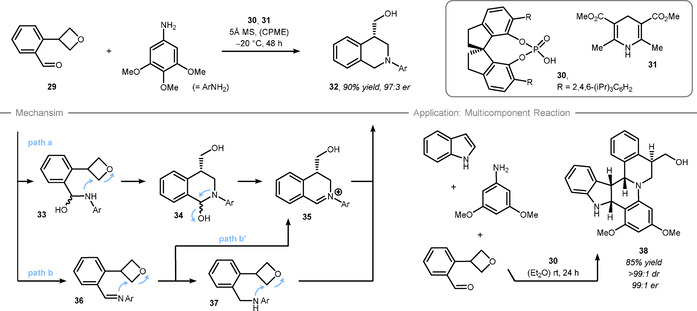
Sun's mechanistic proposal for the tetrahydroisoquinoline formation. CPME = cyclopentylmethylether. dr = diastereomeric ratio.

Regarding nitrogen nucleophiles, ring‐opening reactions of oxetanes are challenging indicated by the harsh conditions used in early studies by Ziemann and Gregory.^[30]^ More recent reports are typically based on an intramolecular approach targeting heterocyclic rings. In this regard, the Kuduk and Steward groups reported one‐pot procedures that attach 3‐aminooxetane to the aromatic scaffold followed by a subsequent ring‐opening reaction to forge N‐heterocycles such as quinazolines or indazoles in a racemic fashion.^[31]^ In terms of enantioselectivity, the Sun group reported the synthesis of tetrahydroisoquinolines through desymmetrization of 2‐oxetanylbenzaldehyde **29** in 2013 (Scheme 9, top).^[32]^ This one‐pot procedure is high yielding and highly enantioselective, although the reaction is limited to electron rich aryl amines. To carry out this formal reduction, the addition of Hantzsch ester **31** was required. The authors suggest two possible reaction pathways (Scheme 9, bottom left). In path a, the reaction proceeds via *N,O*‐hemiacetal **33**, which nucleophilically attacks the oxetane motif to form intermediate **34**. Dehydration provides iminium **35**, which gets reduced to tetrahydroisoquinoline **32**. Alternatively, a reductive amination via intermediate **36** takes place providing amine **37**, which opens the oxetane ring to furnish product **32** (path b). It is also possible that imine **36** undergoes nucleophilic ring‐opening to intermediate **35** (path b’). Further studies on the activation mode of this Brønsted acid catalyzed reaction by Houk et al. suggested that the oxetane‐ring and the nitrogen nucleophile are both activated by the Brønsted‐acidic and Brønsted‐basic sites of the phosphoric acid **30** (cf. Figure 1).^[21]^ To showcase the synthetic applicability, the Zhu and Sun groups applied this strategy in a multi‐component aza‐Diels–Alder reaction with indoles to form highly complex polycyclic indolines such as **38** in good diastereo‐ and enantioselectivity (Scheme 9, bottom right).^[33]^

**Scheme 10:**
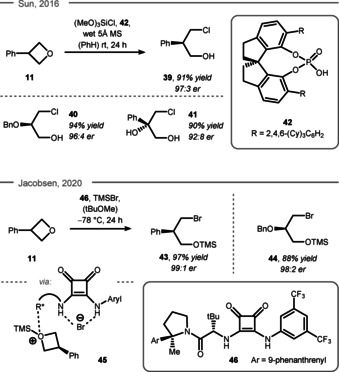
Top: Sun's HCl‐based desymmetrization of oxetanes. Bottom: Jacobsen's 1,3 bromohydrin synthesis. Cy = cyclohexyl.

Besides the common nucleophiles oxygen, sulfur, carbon, and nitrogen, desymmetrization of oxetanes has been achieved with phosphorus‐species in context of ligand design.^[34]^ Despite the importance of chiral phosphines as ligands, no enantioselective variant has been reported so far. Furthermore, halogens are known to undergo nucleophilic attack at oxetane rings.^[35]^ An enantioselective variant of this ring‐opening reaction was reported by Sun et al. using CPA **42** for enantioinduction (Scheme 10, top).^[36]^ To this end, a wide range of substituents on the oxetane ring were employed (e.g. phenyl, **11** to **39** and benzylether, **40**) as well as 3,3‐disubstitution, which gave rise to fully substituted stereocenters in a stereocontrolled fashion (e.g. **41**). Mechanistic experiments omitting CPA **42** showed effective background reaction, which made optimization of the reaction parameters particularly challenging. The key to success was the small and continuous release of water from wet molecular sieves, which triggered the continuous release of HCl (via the reaction of water with trimethoxy silyl chloride) and allowed the ring‐opening to occur in a highly enantioselective fashion. To get further insight into this reaction, theoretical calculations were performed confirming a bifunctional activation mode of the CPA through coordination of its Lewis acidic side to the oxetane ring and its Lewis basic side to the HCl (cf. Figure 1).^[23a]^

An elegant ring‐opening with bromide as nucleophile was reported by Jacobsen and co‐workers in 2020 (Scheme 10, bottom).^[37]^ Trimethylsilyl bromide acted as the bromide source, which enhanced the electrophilicity of the oxetane through silylation of the Lewis basic oxygen and allowed the formation of bromo ether **43** from phenyloxetane **11**. Functional groups such as benzyl ether **44** were also compatible with this reaction protocol. Mechanistically, the chiral squaramidic catalyst **46** was proposed to interact with the bromide via its hydrogen bonding motif and with the substrate through its Lewis basic amine functionality (see structure **45**). Thus, excellent enantioselectivity was achieved for the delivery of the bromide to the oxetane. Kinetic isotope effect (KIE) analysis indicated that silylation of the oxetane ring was reversible and the bromide ring‐opening/C−O bond cleavage enantiodetermining.

## 3 Remote Oxetane Desymmetrization

**Scheme 11:**
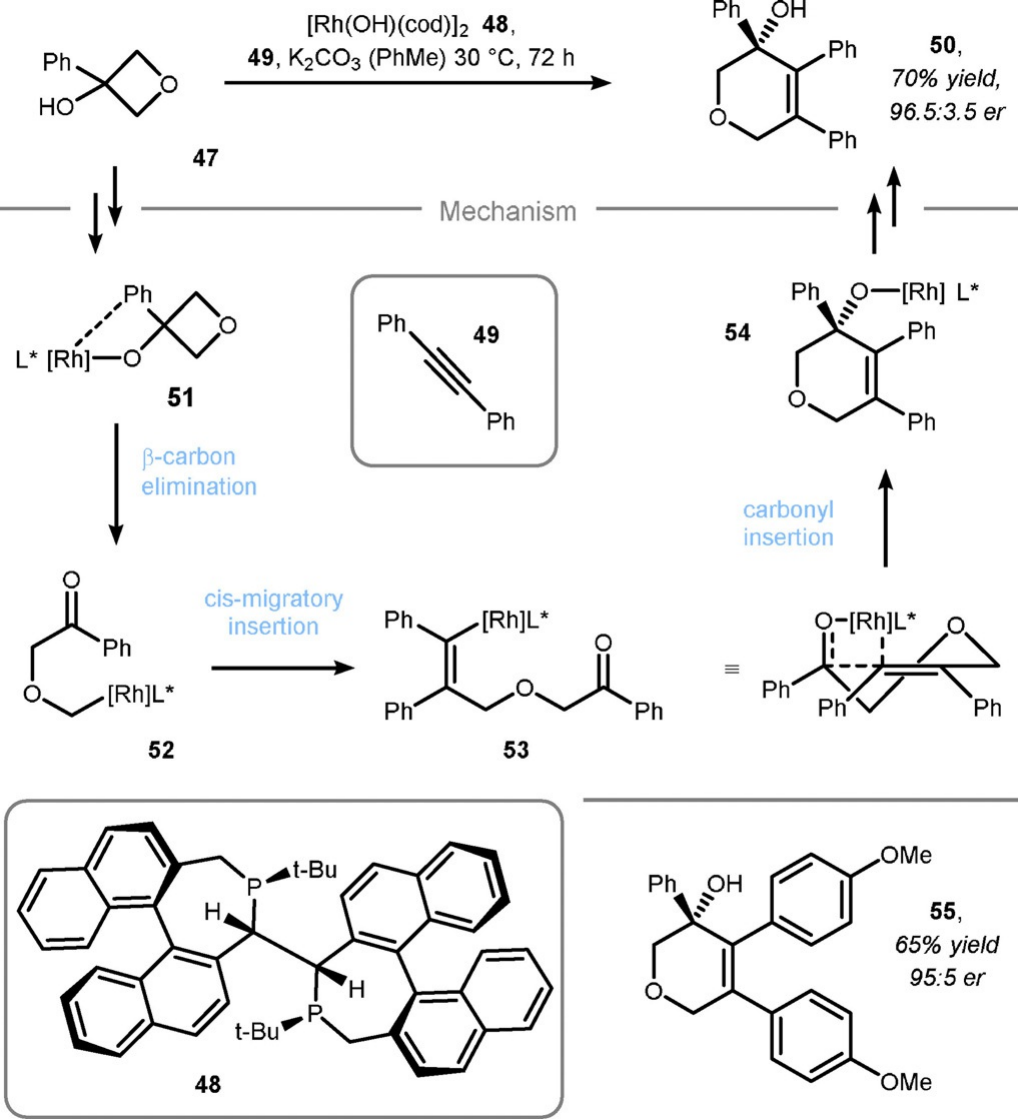
Rhodium‐catalyzed ring‐expansion of oxetanes by Zhang.

Besides Lewis acids and Brønsted acids, desymmetrization reactions of oxetanes can be promoted by transition metal catalysts activating the remote C−C bond. In this context, Zhang et al. reported a ring expansion reaction of 3,3‐disubstituted oxetane **47** with a rhodium catalyst and alkyne **49** (Scheme 11).^[38]^ The reaction likely proceeds via the coordination of the catalyst to the hydroxy and the aryl moiety of **47** (intermediate **51**). After subsequent β‐carbon elimination to intermediate **52**, migratory insertion across the alkyne followed by ring‐closure and protoderhodation (from **54**) gives rise to cyclic ether **50**. The geometry of intermediate **53** determines the stereoisomeric outcome of the reaction, which was induced by BINAPINE ligand **48**. Other symmetric alkynes were also viable in this synthetic sequence (e.g. **55**). A related, racemic example of a ring‐expansion reaction was reported by Miura et al., in which a C−H‐activation strategy was combined with the ring‐opening and closing reaction of the oxetane.^[39]^

**Scheme 12:**
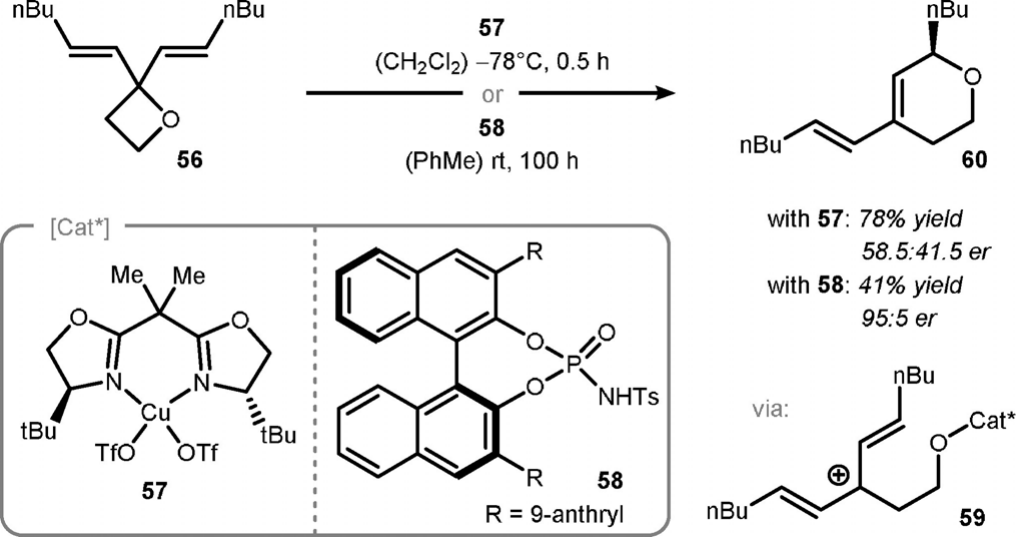
Njardarson's desymmetrization of 2,2‐disubstituted oxetane **56** with a Cu^II^‐ or phosphoramidate catalyst.

Desymmetrization reactions based on 2,2‐disubstitution at the oxetane ring are less studied and there is only one report by the group of Njardarson to date.^[40]^ In this study, 2‐monosubstituted oxetanes were the primary object of investigation. However, to explore the mechanism the authors subjected symmetric divinyl oxetane **56** to their optimal reaction conditions (Scheme 12). It was suspected that the reaction proceeds through a stepwise mechanism, where the copper is activating the oxetane as a Lewis acid, followed by ring‐opening. The cation **59** is delocalized through the allylic system and by a nucleophilic attack of the oxygen atom, the oxolane ring is formed. Higher activity of copper‐catalyst **57** compared to the phosphoramidate **58** was observed with a contradictory influence on the enantioselectivity of the product **60**.

Remote desymmetrization of oxetanes can also be achieved through rearrangement. In this context, semipinacol rearrangements are particularly popular, but are typically performed on cyclobutanol ring expansions.^[41]^

**Scheme 13:**
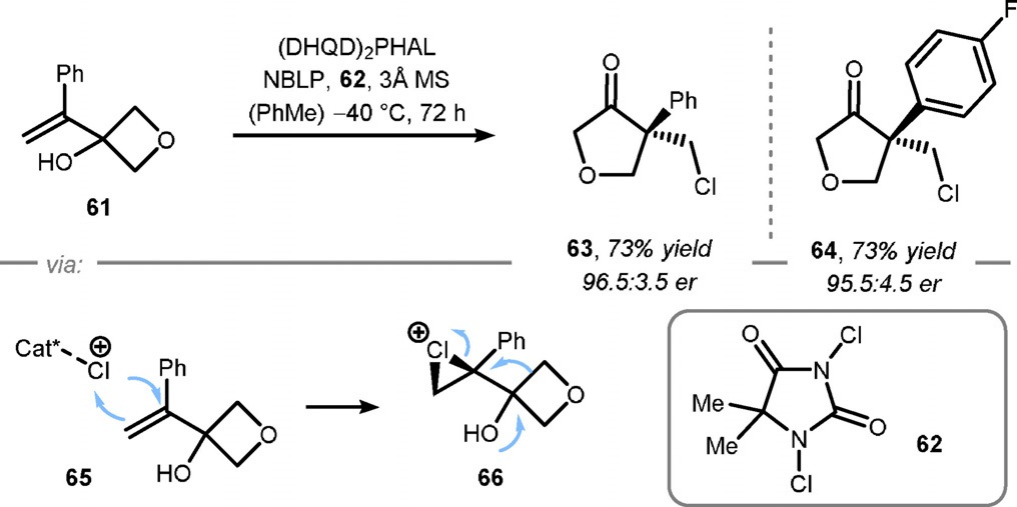
Asymmetric semipinacol‐rearrangement by You. NBLP = *N*‐tertbutyloxycarbonyl‐l‐phenylgylcine.

Regarding oxetanes, the You group recently investigated the behavior of oxetane derivatives in such a rearrangement reaction (Scheme 13).^[42]^ Therefore, allylic alcohol **61** was submitted to a reaction sequence based on electrophilic chlorine source **62**. The reaction likely proceeds via the enantioselective formation of chloronium **66** from the addition of an electrophilic chlorine‐atom to the C−C double bond of the allylic alcohol **61**. Chloronium **66** can then undergo a semipinacol rearrangement generating cyclic ketone **63** and **64**, respectively. The addition of *N*‐Boc‐(L)‐phenylglycine (NBLP) and the phthalazine adduct of dihydroquinine ((DHQD)_2_PHAL) was found to be crucial for the enantioinduction in this reaction (presumably via coordination of Cl^+^ before alkene attack, see **65**).

## 4 Conclusion

This review highlights the synthetic utility of prochiral oxetanes, which is based on their inherent ring‐strain and Lewis‐basicity. Elegant desymmetrization strategies have been established for 3‐substituted, 3,3 as well as 2,2‐disubstituted oxetanes, which give rise to a diversity of products. The enantioselectivity of these reactions can be controlled through sophisticated choice of Lewis acids or Brønsted acids, with CPAs being the most widely used catalyst to date. Some of the presented reactions allow control over remote stereocenters, which marks a unique strategy in the synthesis of chiral alcohols and ethers. However, when compared to the synthetic impact of oxiranes (epoxides), oxetanes are still far less studied and their application in synthetic endeavors lags behind. Additionally, the possibility of oxetanes to undergo other reactions than nucleophilic ring‐openings at the α position were only recently explored indicated by three examples from the Zhang, You, and Njardarson groups. A further reason for the lack of application is the easy, but stepwise formation of the oxetane ring that cannot compete with epoxidations. The comparable physical properties of oxetane in terms of ring strain as well as its significance as a bioisoster are however promising and set the basis for future explorations to fully exhaust the immense possibilities of oxetanes for synthesis and pharmaceutical applications. In particular desymmetrization reactions resemble an attractive strategy, as they allow oxetanes to be converted to highly complex scaffolds bearing quaternary (all‐carbon) stereocenters in a step‐economic fashion.

## Conflict of interest

The authors declare no conflict of interest.

## Biographical Information

*Alexander Sandvoß studied chemistry at Westfälische‐Wilhelms Universität Münster as Studienstiftung scholar and at Cardiff University (Prof. T. Wirth) as Erasmus fellow. After a research stay in the group of Prof. A. Studer on on‐surface reactions*, *he graduated in 2019 and joined the Wiest laboratory shortly after. Currently*, *he is working on strain‐mediated reaction development involving cyclic ethers and on applications in their synthesis*.

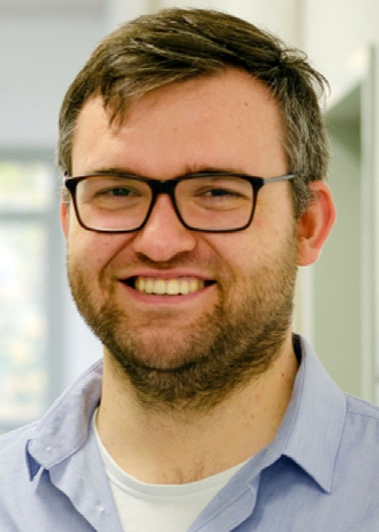

## Biographical Information

*Johannes Wiest attended the Universität Basel*, *Switzerland*, *where he obtained his M.Sc. in 2012. After a research stay at the Scripps Research Institute (Prof. D. G. Blackmond)*, *he moved to Munich to pursue his Ph.D. at the Technische Universität München with Prof. T. Bach. He held a DFG research fellowship at Indiana University (Prof. M. K. Brown) before starting his independent career at the Westfälische‐Wilhelms Universität Münster. Since 2020*, *he is an Assistant Professor at the Johannes Gutenberg‐Universität in Mainz*.